# Not All Kinds of Revegetation Are Created Equal: Revegetation Type Influences Bird Assemblages in Threatened Australian Woodland Ecosystems

**DOI:** 10.1371/journal.pone.0034527

**Published:** 2012-04-06

**Authors:** David B. Lindenmayer, Amanda R. Northrop-Mackie, Rebecca Montague-Drake, Mason Crane, Damian Michael, Sachiko Okada, Philip Gibbons

**Affiliations:** Fenner School of Environment and Society, ARC Centre of Excellence for Environmental Decisions, and National Environment Research Program, The Australian National University, Canberra, Australia; Australian Wildlife Conservancy, Australia

## Abstract

The value for biodiversity of large intact areas of native vegetation is well established. The biodiversity value of regrowth vegetation is also increasingly recognised worldwide. However, there can be different kinds of revegetation that have different origins. Are there differences in the richness and composition of biotic communities in different kinds of revegetation? The answer remains unknown or poorly known in many ecosystems. We examined the conservation value of different kinds of revegetation through a comparative study of birds in 193 sites surveyed over ten years in four growth types located in semi-cleared agricultural areas of south-eastern Australia. These growth types were resprout regrowth, seedling regrowth, plantings, and old growth.

Our investigation produced several key findings: **(1)** Marked differences in the bird assemblages of plantings, resprout regrowth, seedling regrowth, and old growth. **(2)** Differences in the number of species detected significantly more often in the different growth types; 29 species for plantings, 25 for seedling regrowth, 20 for resprout regrowth, and 15 for old growth. **(3)** Many bird species of conservation concern were significantly more often recorded in resprout regrowth, seedling regrowth or plantings but no species of conservation concern were recorded most often in old growth. We suggest that differences in bird occurrence among different growth types are likely to be strongly associated with growth-type differences in stand structural complexity.

Our findings suggest a range of vegetation growth types are likely to be required in a given farmland area to support the diverse array of bird species that have the potential to occur in Australian temperate woodland ecosystems. Our results also highlight the inherent conservation value of regrowth woodland and suggest that current policies which allow it to be cleared or thinned need to be carefully re-examined.

## Introduction

Much has been written in many parts of the world about the value of regrowth vegetation, including its importance for biodiversity conservation [1]–[5]. This includes regrowth after logging, after vegetation clearing, and after agricultural land abandonment. Indeed, ecological studies of regrowth vegetation have been a classic part of ecology for a long time [6] and a wide range of studies from around the world have demonstrated that regrowth vegetation can be important for a range of species. This is true both in tropical and temperate ecosystems (e.g. [4], [7]–[13]), although such kinds of vegetation can often support different suites of species compared with, for example, old growth vegetation [4], [14]–[16].

While the value of regrowth vegetation is increasingly recognised, there can be different kinds of revegetation that have different origins. That is, different starting conditions and/or disturbance regimes can give rise to structurally different kinds of revegetation. For example, in the threatened temperate woodlands of southern Australia (where the study we report here is taking place), different kinds of regrowth vegetation can include: **(1)** resprout regrowth vegetation which develops following logging, fire or partial clearing, e.g. [17], and **(2)** seedling regrowth which develops after a reduction in grazing pressure by domestic livestock [18] or after some kinds of disturbance like ploughing after droughts. In addition, throughout threatened Australian temperate woodlands, there are extensive efforts to replant native vegetation [19]–[22]. A key question is: Are there differences in the richness and composition of biotic communities in different kinds of regrowth and how does this compare to old growth and plantings? The answer to this question remains either unknown or poorly known in many ecosystems worldwide. Yet it is critical to know whether different kinds of restoration efforts like the deliberate planting of vegetation or the natural regeneration of vegetation lead to the development of different suites of species that are associated with them. This is, in part, because the costs of deliberately planting vegetation can be very high but those associated with passive revegetation (i.e. resprout and seedling regrowth) can be comparatively much lower [21]. In addition, some authors, e.g. [23] have argued that biodiversity has been negatively affected in some replanted areas because stem density has been too high relative to that typical of passively regenerating areas. For example, this may impede foraging by bats and slow the rate of development of key structures like large cavity trees [24].

We addressed key knowledge gaps about the conservation value of different kinds of revegetation in the temperate woodlands ecosystems of southern New South Wales, south-eastern Australia. Temperate woodlands are some of the most heavily cleared, extensively degraded, and highly threatened ecosystems on the Australian continent [25] and there is an urgent need for vegetation restoration in many areas [18]. However, to the best of our collective knowledge, no-one has previously compared the biodiversity value of different kinds of revegetation. We focused on birds in our comparative study of growth types. This was because: **(1)** there is a wide range of bird species of conservation concern in Australian temperate woodlands [26], [27], **(2)** past work has indicated that some elements of the temperate woodland bird biota respond strongly to key attributes of stand structure (e.g. [11], [28]) which are likely, in turn, to vary substantially between different kinds of revegetation, and **(3)** birds are widely considered to play important roles in maintaining some ecosystem processes [29].

Using an extensive dataset gathered at a large number of sites (N = 193) that have been surveyed repeatedly over the past ten years, we posed the following series of broad and inter-connected questions:

- Is there a difference in the bird species richness and the composition of bird assemblages between old growth temperate woodland and different kinds of revegetation, including plantings? We postulated at the outset of this study that there would be marked differences in bird species richness and assemblage composition between old growth, resprout regrowth, seedling regrowth and planted areas. This was because of likely major differences in vegetation structure between growth types and previously well documented relationships between vegetation structure and bird responses as reflected through ecological theories like the structural complexity hypothesis [30], the intermediate disturbance hypothesis [31], and the biological legacies concept [32]. In addition, there are marked differences in starting conditions between types of revegetation and this also can influence biotic responses [33].
- Are there bird species associated with particular growth types? Guided by theory like the landscape texture hypothesis [34], [35], at the onset of this investigation we predicted that small-bodied birds would be closely associated with densely structured revegetated areas [36], particularly plantings. Based on succession theory [37], we predicted that particular bird taxa like cavity-dependent species would be strongly associated with old growth where vegetation attributes like trees with hollows are likely to be most abundant.

Notably, we elected to make our investigation a comparative study of broad categories of growth types, rather than focus on relationships between birds and an array of covariates corresponding to measurements of vegetation structure and plant species composition, e.g. [11], [28], [38]. We made this decision because the vast majority of on-ground practitioners in south-eastern Australia charged with managing native vegetation readily recognise broad growth type categories (i.e. old growth, resprout regrowth, seedling regrowth, plantings). Moreover, such broad categories are a fundamental part of government legislation such as in Queensland and New South Wales [39]. Conversely, few practitioners have the time or expertise to complete detailed measurements of stand structure and composition and then relate them to response variables like bird species richness or the presence of individual bird species.

Addressing questions about the conservation value of different kinds of revegetation is important for several key reasons. First, approximately 40% of the planet's terrestrial land surface is used being used for agriculture [40], [41] but 16–40% of that area is lightly to severely degraded and in need of some form of restoration [42]. Second, because there can be substantial differences in the costs and labour requirements of different forms of revegetation (e.g. replanting versus passive regeneration) [21], it is critical to better understand the value of different areas as habitat for wildlife. Third, there has been extensive clearing of regrowth vegetation in some parts of the world (e.g. eastern Australia) [17], [43]. Fourth, there are well advanced proposals to undertake management interventions like thinning in large areas of regrowth vegetation, including in many areas of threatened temperate woodland in Australia [44]. This activity is hypothesized to increase pasture growth for livestock grazing or to increase the rate of diameter increment of overstorey trees and thereby accelerate the pace at which large trees (and associated key attributes like cavities and large pieces of coarse woody debris) will develop. However, practices like thinning need to be guided by an understanding of the value of different kinds of regrowth for biodiversity.

## Methods

### 1.1 Study area

**Figure 1:**
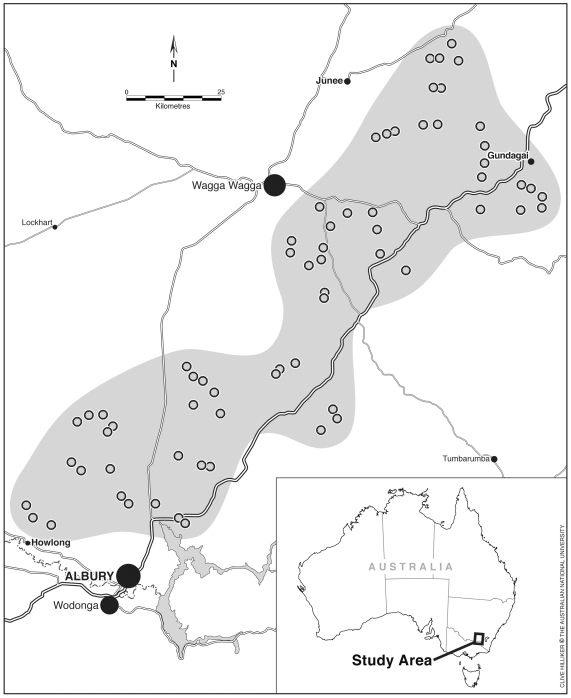
The South-west Slopes study region of southern New South Wales, south-eastern Australia.

We conducted this study within the South-west Slopes region of New South Wales in an area spanning the towns of Junee (0552952 E 6140128 N) in the north and Albury (0494981 E 6008873 N) in the south (a distance of ∼150 km), and Gundagai (600532 E 6119073 N) and Howlong (467090 E 6017897 N) in the east and west respectively (a distance of ∼120 km) (Figure 1). The predominant form of native vegetation in the region is temperate woodland [25], [45] dominated by White Box *Eucalyptus albens*, Grey Box *E. microcarpa*, or other eucalypt tree species such as Yellow Box *E. melliodora*, Blakely's Red Gum *E. blakelyi*, Red Stringybark *E. macrorhyncha* and Red Ironbark *E. sideroxylon*. A range of broad vegetation types has been recognised in our study area [46] and these are Floodplain Transition Woodlands, Inland Riverine Forests, Upper Riverina Dry Sclerophyll Forests and Western Slopes Grassy Woodlands.

### 1.2 Study design

Our study comprised 193 sites on 46 individual farms that we assigned to one of four vegetation growth types plantings (63 sites), old growth woodland (71 sites), resprout regrowth woodland (27 sites), and seedling regrowth woodland (32 sites). Plantings were areas of planted native vegetation characterised by a mix of local endemic and exotic Australian ground cover, understorey and overstorey plant species. Most plants were typically spaced 2 m apart, but there was not a standard set of spacing and plant species composition protocols applied in revegetation efforts. All plantings were at least 7 years old at the start of this investigation in 2000 and many were 10–20 years old and had been established to mitigate problems associated with soil erosion and/or salinity. Resprout regrowth refers to (multi-stemmed) regrowth from existing living trees recovering after disturbance by fire, clearing or both. Seedling regrowth originates from seeds germinating after being dropped by overstorey trees. As in the case of our plantings sites, the stands of resprout regrowth and seedling regrowth that we selected exceeded 7–10 years old (when we commenced our work in 2000) but many were 15–20 years old. Old growth woodland was dominated by large old scattered trees, typically 200 or more years old. Livestock grazing occurred in many of our study sites and some were in a degraded condition as a result, particularly where high-intensity set stock grazing regimes were employed. However, there also were sites in good condition in all growth types, for example, those on farms subject to cell grazing and/or short-rotation rotational grazing.

Critically for our study design, our resprout regrowth, seedling regrowth and replanting sites were approximately the same age, whereas the old growth stands were clearly much older. In addition, we worked hard to ensure that the size of patches of old growth, resprout regrowth, seedling regrowth and replanting were broadly similar to avoid the potential for confounding between patch size and growth type; see [9].

### 1.3 Site establishment

We established a permanent fixed 200 m long transect at each of our 193 sites. The permanent transect was where we completed counts of birds (see below) as well as completed detailed measurements of vegetation composition (see Appendix S1).

No specific permits were required for the described field studies. The relevant permissions to enter the private land involved in the study were given by the Mr Emmo Willinck, Catchment Officer, on behalf of the Murray Catchment Management Authority (MCMA). No specific permits were required for the field study locations as the owners of the private land involved had established access relationships with the MCMA. The researchers were acting as agents for the MCMA under the terms of a collaborative research partnership. However, prior to all surveys, telephone contact was made with all the relevant private landowners to indicate researcher access to their land. All native animal species and native woodland vegetation are protected in Australia, including endangered birds and plants. Our studies were observational investigations and no plants or animals were harmed in any way.

### 1.4 Bird counting protocols

We gathered data on temperate woodland birds at all 193 sites in our investigation between 2002 and 2009. These data comprised five spring counts and three winter counts. Thus, we surveyed each of the 193 sites eight times for birds between 2002 and 2009. Our bird counting protocols entailed repeated 5 minute point interval counts [47] at the 0 m, 100 m and 200 m points along the permanent transect at each site. Six highly experienced ornithologists participated in the surveys although they varied to some extent in their ability to detect some groups of birds. Lindenmayer et al. [48] showed that pooling counts of two or more observers at the same site could compensate for extra variability due to observer heterogeneity. Field et al. [49] showed that weather and other conditions on any given day can influence bird detectability. Thus, in each of our surveys, each permanent field site was surveyed by two different observers on different days by repeated point interval counts. We completed counts between 5.30–9.30am and did not undertake surveys on days of poor weather (rain, high wind, fog or heavy cloud cover). These protocols were identical to those employed in other long-term major studies in woodlands [11], [46], [50]. In summary, between 2002 and 2009, we conducted 48 individual point counts at each of our 193 permanent field sites.

### 1.5 Data aggregation

Our approach provided high quality presence-absence data. There were six opportunities for a given bird species to be detected within a particular site in any given survey period (i.e. 3 point counts completed by 2 observers in a given survey). This was then summarised as a single presence/absence value for a particular bird species at a site, in a particular season, in a particular year.

The species we targeted for investigation were readily recognisable and had distinctive calls. Moreover, we were extremely familiar with them from many previous studies in a range of environments in south-eastern Australia [20], [51], [52]. Tyre et al. [53] found that six repeated visits at a site improved the precision of estimates to levels comparable to that achieved with conventional statistics in the absence of false-negative errors. As noted above, our dataset comprised 48 point counts completed during eight surveys between 2002 and 2009. Furthermore, our preliminary data analyses revealed very few newly detected species (<2%) by our spring (2006) and winter (2007) surveys. Moreover, for our study, “plots” were sampling units rather than specific territories for which it would be appropriate to determine true occupancy. We therefore assumed that non-detection was low for the bird species included in this study. Moreover, although some individual birds may have gone undetected, this would not invalidate a comparative investigation like ours.

In summary, the work we report in this paper was a comparative study in which we were interested in quantifying differences in the bird biota of different growth types. Therefore, a key statistical underpinning is that we have used an identical field counting methodology with the same observers surveying the same sites consistently in successive surveys. The currency that we have applied in our comparative study is presence/absence.

### 1.6 Vegetation measurements

We completed detailed measurements of the vegetation at each of our 193 field sites to enable us to compare the vegetation structure of the different growth types (see below). First, we established a 20×20 m plot around the 0 m, 100 m and 200 m post at each site. Second, at each corner of each 20×20 m plot, we established a 1×1 m plot. This gave 3 large (20 m×20 m plots) and 12 1 a 1 m plots at each site. The array of vegetation attributes measured at each site is described in Appendix S1.

### 1.7 Statistical analysis

First, we analysed relationships between bird species richness and growth type using repeated measures MANOVA. For the analysis, a particular site was the subject, the between-subject effect was growth type, and the within- subject effect was time.

Second, we explored relationships between the composition of the bird assemblage and the growth type using both partial Canonical Correspondence Analysis (CCA) and partial redundancy analysis. We conducted these analyses using the package “vegan” in program R. As the results from both were similar, we elected to present only those results from CCA, which is apposite when analysing occurrence data as it detects patterns within ecological datasets that can be explained by environmental variables [54]. We used the CCA algorithm developed by Legendre and Legendre [55] to analyse relationships between bird species occurrences and growth type. Constraining variables were subjected to weighted linear regression and a correspondence analysis was conducted on the fitted values via singular value decomposition. We controlled for the effects of season and year by including them as covariates in the partial CCA [55]. We used Monte Carlo simulations, with 1000 steps, to establish the significance of all canonical axes. Tests of significance in CCA do not rely on parametric assumptions [56], [57]. CCA allows a visual interpretation (a biplot) of species-environment relationships. We used species conditional scaling [58] to centre species within the sites in which they occurred. Distances between species and a particular growth type approximate their relative frequency of occurrence; see [59]. We restricted the data used in the CCA to bird species that were detected in five or more sites on average over the ten year period. This was because rare species can obscure community patterns [57], [60].

Third, we examined the response of individual bird species to growth type. Some of these birds were taxa of conservation concern [11], [38] and others, like the hyper-aggressive native honeyeater Noisy Miner (*Manorina melanocephala*), are known to exclude a range of other species from temperate woodland [61]. To quantify relationships between growth types and individual bird species, we conducted Cochran-Mantel-Haenszel tests [62] which were stratified by time. We then used Analysis of Means (ANOM) of proportions [63] to determine if occurrences of bird species in a particular growth type were significantly higher or lower than the average occurrences in all growth types.

Finally, we postulated that the underlying drivers of growth type differences in bird assemblages may have been a result of differences in key attributes of vegetation structure. On this basis, we explored differences in vegetation structure of the growth types to better understand the potential reasons for species' preferences. We employed a nonparametric Steel-Dwass multiple comparison procedure [64]–[66] to test for differences in the structure of different growth types.

## Results

We recorded 178 species of birds in our dataset that was comprised of 9264 survey points. Of these, we detected 57 species of birds at fewer than four sites on average per year, and excluded them from subsequent data analyses. Our focus was on woodland birds, and we therefore removed 31 waterbirds from our analysis. This left a total 90 species for detailed investigation. We list the common and scientific names of these species in Appendix S2.

### 2.1 Growth type and bird species richness

**Figure 2:**
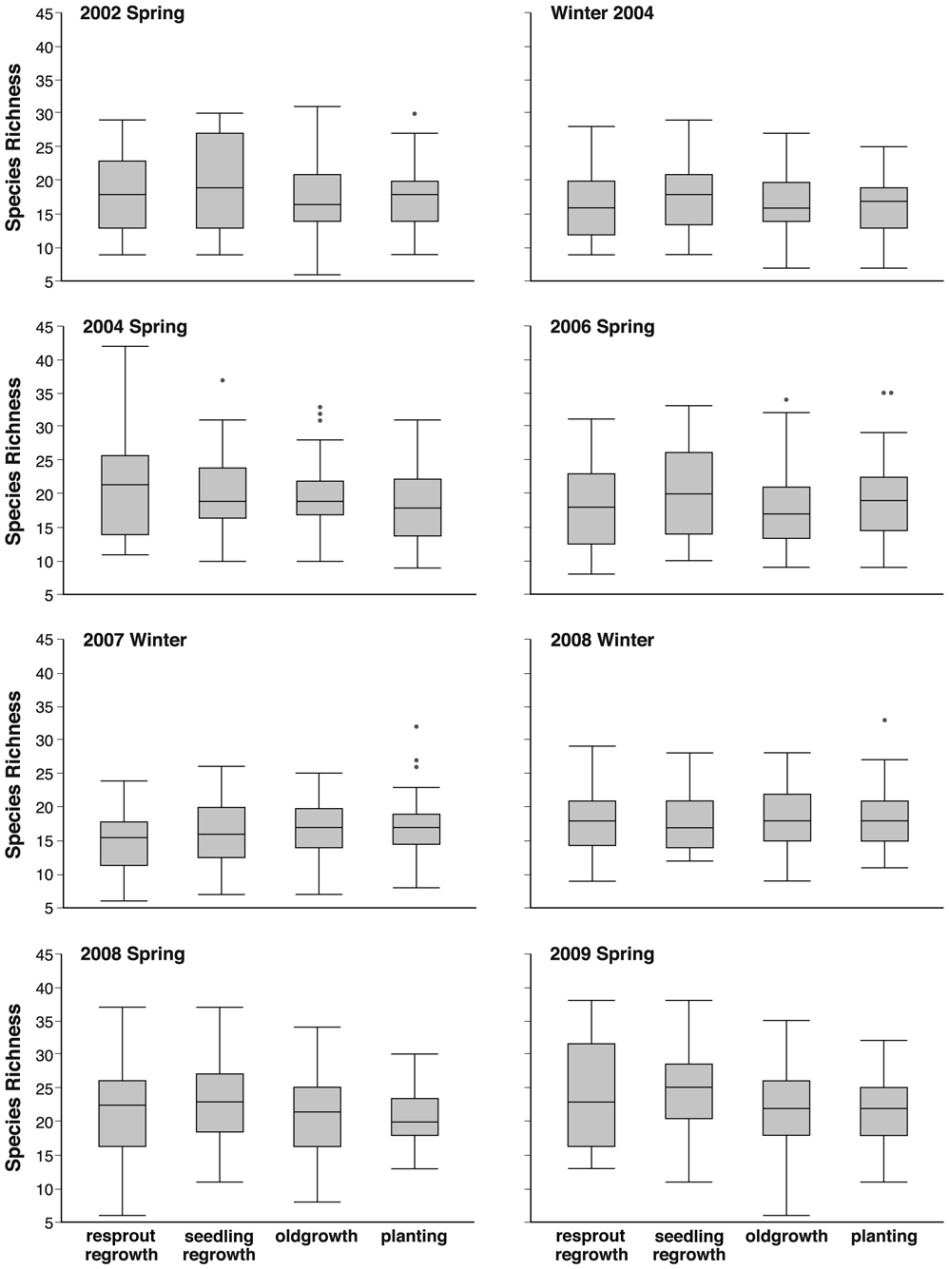
Average counts for growth types in a particular year and season.

Our repeated measures MANOVA showed insufficient evidence overall to conclude there were significant differences in species richness between the various growth types (F-test_3,159_ = 0.824, p-value = 0.4822) (Figure 2).

### 2.2 Growth type and the composition of the bird assemblage

**Figure 3:**
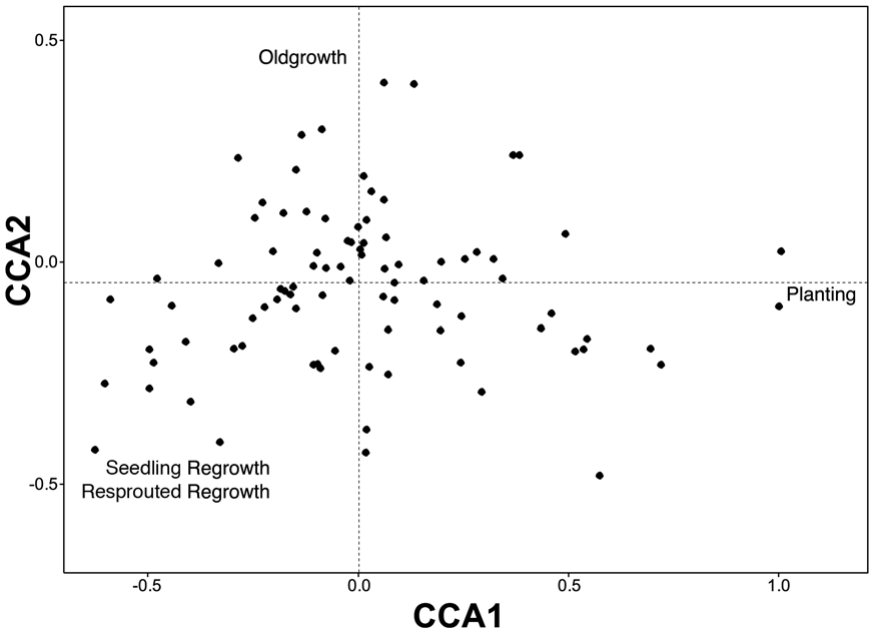
Biplot of the first two canonical axes showing species and growth types. Distances between species approximate the chi-squared distance between species distributions (see [59] for details of the approach used in data analyses).

**Figure 4:**
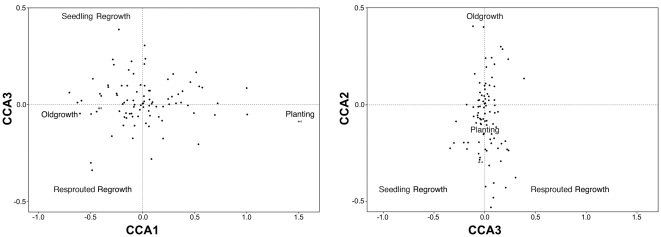
Correspondence analysis biplots of bird species and growth type. The diagrams are: (left) first versus third dimensions from correspondence analysis and (right) second and third dimensions from correspondence analysis. Distances between species approximate the chi-squared distance between species distributions (see [59] for details of the approach used in data analyses).

We found that the first three axes of the CCA were statistically significant using 1000 permutations (P<0.05). The first axis accounted for 72% of the constrained variation in our data and it contrasted occurrences of bird species in plantings from the other three growth types (Figure 3). The second axis accounted for 22% of the constrained variation, and contrasted old growth from the two types of regrowth (Figure 3). The third axis, which accounted for the least amount of variation, contrasted resprout regrowth and seedling regrowth (Figure 4).

### 2.3 Individual species responses

We found that 29 species occurred significantly more often in plantings, 25 significantly more often seedling regrowth, 20 significantly more often in resprout regrowth, and 15 significantly more often in old growth (Appendix S2). Of the 90 species for which we completed detailed statistical analyses, 67 exhibited a significant (P<0.05) relationship with growth type (Appendix S2). A number of these were species of conservation concern. For example, the Grey-crowned Babbler and White-browed Babbler were significantly more likely to occur in seedling regrowth than other growth types (Appendix S2). The Black-chinned Honeyeater was most often found in seedling regrowth and resprout regrowth. In the case of the Diamond Firetail, the species occurred significantly less often in old growth relative to other growth types (Appendix S2). The Hooded Robin was significantly more likely to occur in resprout regrowth than other growth types, particularly plantings. The Brown Treecreeper, Crested Shrike-tit, Dusky Woodswallow and Jacky Winter were least likely to be recorded in plantings and most likely to be recorded in resprout regrowth and seedling regrowth. Birds of conservation concern that were most often recorded in plantings included the Red-capped Robin, Rufous Whistler, Speckled Warbler, and Flame Robin (Appendix S2). Notably, no birds of conservation concern were found the majority of the time in old growth (Appendix S2).

### 2.4 Vegetation structure and growth type

**Figure 5:**
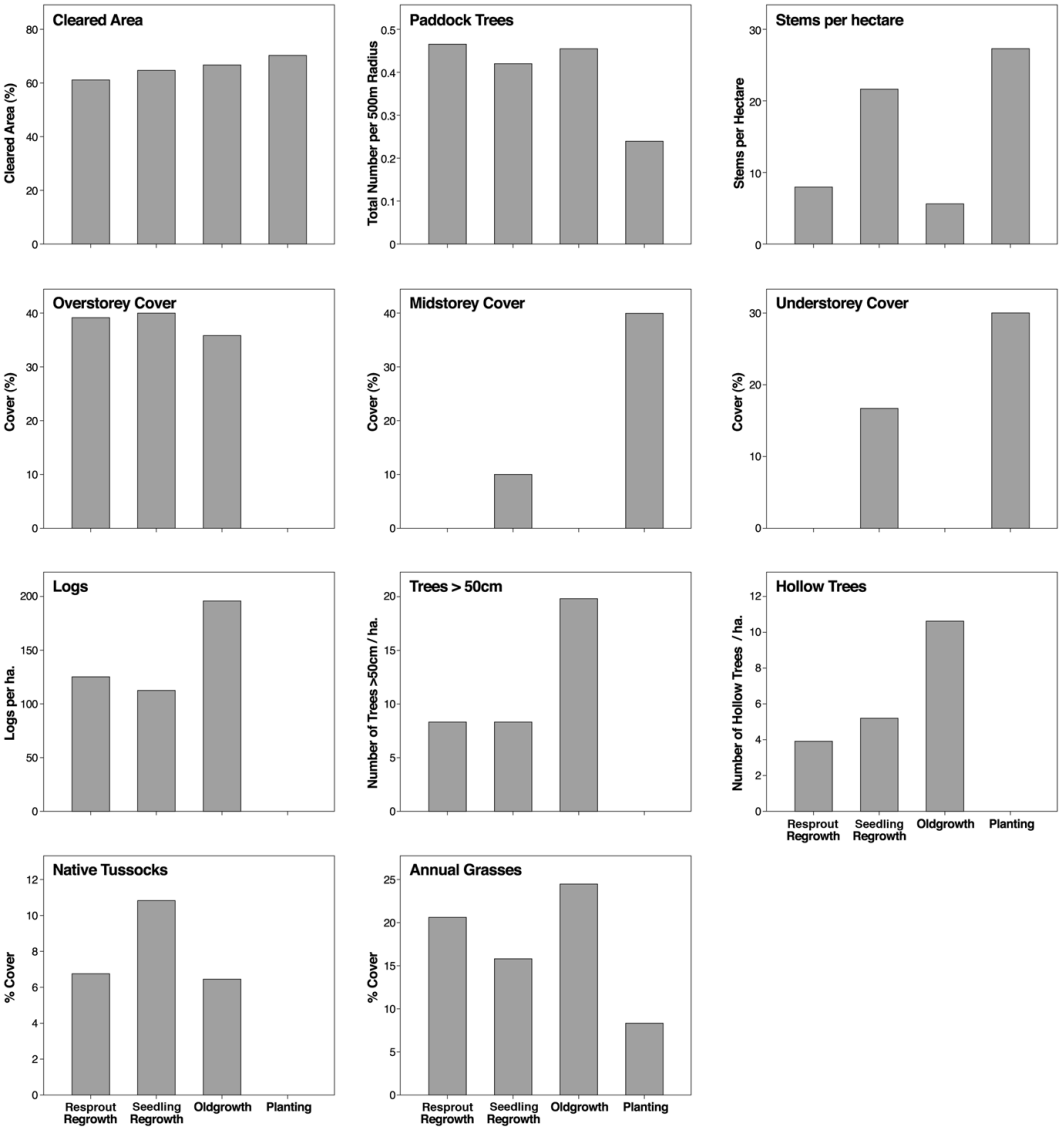
Median values for vegetation characteristics in different growth types.

We completed analyses of the structure of vegetation of the four growth types using Steel-Dwass multiple comparison tests (Appendix S3). These analyses clearly indicated marked growth type differences in vegetation structure (Figure 5; Appendix S3). For example, we found that plantings had significantly higher stem density than old growth and resprout regrowth. It also had significantly higher midstorey cover and significantly lower overstorey cover than other growth types (Figure 4; Appendix S3). In contrast, old growth was characterised by significantly more trees with hollows, large logs and mistletoe than plantings and both seedling regrowth and resprout regrowth (Appendix S3).

## Discussion

We have compared the bird assemblages of different growth types in the temperate woodlands of south-eastern Australia using a dataset comprised of a large number of sites that have been surveyed numerous times over the past decade. Many past studies have examined the biodiversity values of particular kinds of temperate vegetation such as old growth woodland [11], regrowth [17] or plantings [19], [20]–[22]. However, to the best of our collective knowledge, and to the knowledge of a range of colleagues whom we contacted about this paper (see Acknowledgments), no-one in Australia has completed a comparative study of the bird biota of different growth types of temperate woodland, including different kinds of passively regenerated regrowth as well as plantings. For example, while the biodiversity values of resprout regrowth vegetation have been comparatively well studied in Europe [2], [67] and North America [68], a similar level of understanding is generally lacking in Australia (although see [1], [13], [17]).

Our investigation produced a number of key findings. Two key ones were: **(1)** Marked differences in the bird assemblages of resprout regrowth, seedling regrowth, old growth and plantings. **(2)** Many bird species of conservation concern being significantly (P<0.05) more often recorded in resprout regrowth, seedling regrowth or plantings but a paucity of species of conservation concern being recorded most often in old growth. We further discuss these findings in further detail in the remainder of the paper and conclude with some of the key implications for biodiversity conservation and woodland management.

### 3.1 Differences in bird responses to growth types

We found highly significant differences in the bird assemblages of old growth and regrowth temperate woodland and plantings. We also found that many species of conservation concern were associated with resprout regrowth and/or seedling regrowth. A good example was the Hooded Robin which was strongly associated with resprout regrowth woodland – a result consistent with another recent study [69]. We suggest that differences in bird occurrence among different growth types are likely to be strongly associated with growth-type differences in stand structural complexity [24], [70] such as stem density and the prevalence of tree hollows and logs (see Appendix S3). For example, we found that old growth woodland supported significantly higher numbers of trees with hollows than the other growth types that we examined (Appendix S3). This may explain the significantly higher number of recordings of cavity-dependent birds like the Galah, Sulphur-crested Cockatoo, Eastern Rosella and Laughing Kookaburra in this growth type (Appendix S2). Similarly, the paucity of mistletoe in plantings (Appendix S3) may explain the rarity of species closely associated with this resource like the Mistletoebird (Appendix S2). High levels of stem density in plantings and seedling regrowth may explain the prevalence of species such as the Speckled Warbler and the Eastern Yellow Robin, which are of conservation concern. However, multiple factors are likely to influence the occurrence of some bird species in particular growth types. For example, although logs were most abundant within old growth stands (Appendix S3), species such as the Brown Treecreeper, which are often closely associated with this resource [11], [71], [72], were more often recorded in resprout regrowth and seedling regrowth (Appendix S2). The Brown Treecreeper is an insectivore and differences in invertebrate assemblages between growth types [73] are also likely to influence many species of birds, particularly those that are insectivores or partially insectivorous.

One of our unexpected findings was that no species of conservation concern were found significantly more often in old growth (Appendix S2). The reasons for this result are unclear, but it is possible that this result is associated with the fact that old growth temperate woodland in these landscapes was often in a degraded condition [74], [75] as a result of high-intensity grazing by livestock and weed invasion, thereby making these areas unsuitable for a range of species. In addition, the paucity of birds of conservation concern in old growth might be associated with the presence of the hyper-aggressive Noisy Miner (*Manorina melanocephala*) in these areas. This species often excludes smaller species of birds in temperate woodlands [61] and be common in degraded woodland, especially on high productivity areas [76] such as those subject to extensive clearing and over-grazing by domestic livestock. Conversely, the Noisy Miner can be uncommon or even absent from plantings characterized by a dense understory [20] as well as from densely stocked regrowth vegetation. Indeed, our data (Appendix S2) indicated that the Noisy Miner occurred significantly less often in plantings than other growth types (P<0.001) and significantly more often in old growth than other growth types (P<0.001). Hence, the prevalence of the Noisy Miner in old growth woodland might be one of key the factors contributing to the paucity of birds of conservation concern in those areas.

### 3.2 The value of replanted areas

A key result of our study was quantification of the value of plantings for a number of bird species (Appendix S2). Perhaps most importantly, our extensive empirical data indicated that plantings supported several bird species of conservation concern. These included the Flame Robin and the Speckled Warbler. The Red-capped Robin also was found to be closely associated with plantings (Appendix S2), corroborating the findings of Major et al. [77], [78], which showed that areas with a high stem density (e.g. dense shrubs and young saplings; see Appendix S3) were important nest sites for the species. Notably, other work on bird breeding success (that will be reported elsewhere; S Bond et al., unpublished data) has indicated that a number of species of conservation concern (e.g. Speckled Warbler, Southern Whiteface) successfully breed in planted areas. Our findings for birds of conservation concern therefore contrast markedly with those of other workers [22], [79] who have suggested that the biodiversity value of plantings is marginal and such areas are suitable largely for taxa tolerant of disturbed (cleared) open-country landscapes. This, in turn, suggests that the biodiversity values of planted areas may be regionally variable, possibly as a function of factors like the regional amount of potentially suitable habitat.

### 3.3 Management implications

Our study clearly indicates that different growth types of temperate woodland support different assemblages of native birds (Figures 3 and 4). Thus, our findings indicate that it would be inappropriate for bird conservation to clear old growth woodland and replace it with plantings – a conclusion similar to those we have recommended for the mammal and reptile conservation in semi-cleared agricultural landscapes in south-eastern Australia [9]. Rather, our findings suggest that a range of kinds of native vegetation encompassing old growth woodland, regrowth and plantings are likely to be required on a given area of farmland to support the diverse array of bird species that have the potential to occur in temperate woodland ecosystems.

Several recent studies have highlighted how a reduction in grazing pressure by domestic livestock can stimulate the development of seedling regrowth, e.g. [18], [80], [81]. However, it is not always possible for seedling regrowth to establish on some parts of farms such as where there has been long history of clearing and there is no seed bank [82]. In such places, revegetation requires deliberate planting [21]. Indeed, our work strongly indicated that plantings supported a bird assemblage that was significantly different from that of the other growth types (Figures 3 and 4). Different revegetation strategies that are likely to be required on some farms will, in turn, generate different kinds of woodland growth types and support different assemblages of birds.

Of the growth types of temperate woodland that we have examined on farms, areas of resprout and seedling regrowth are those of particular management and conservation concern. This is because while such areas support a range of bird species (including a number of conservation concern; see Appendix S2) and they are an obvious successional stage toward the development of old growth [18], [83], temperate regrowth woodland can nevertheless be partially cleared under existing government legislation such as the *Native Vegetation Act 2003* in New South Wales [39] as well as in Queensland. However, the work we have reported here highlights the importance of regrowth vegetation for a wide range of bird species, including a number of species of conservation concern, as defined by Reid [38]. There is also considerable evidence of the important role that regrowth and other dense woody vegetation plays in key landscape functions such as reducing erosion and the maintenance of the integrity of soil biota [84].

Many landholders are deeply concerned about resprout and seedling regrowth vegetation on their farms, especially in terms of the impacts on grass growth and suitability for grazing as well as potential risks of unplanned fires. Consequently, there has been widespread clearing under the New South Wales *Native Vegetation Act 2003* as well as extensively developed proposals to actively thin resprout and seedling regrowth temperate woodland such as in the prescriptions for the Australian Government's Environmental Stewardship Program [44]. Moreover, several workers have discussed how high-stem-density vegetation may have negative impacts on thermal environments for groups such as reptiles at a local spatial scale [15], [85]. Based on the results of this study, together with other work we (and others) have completed [11], [15], [85], [86], we suggest that thinning must be guided by the habitat requirements and foraging patterns of particular species as well as the management objectives of a given area [87]. The appropriateness of particular management actions in a particular area will depend on which groups of species (e.g. reptiles versus birds), as well as which individual species within a given taxonomic group, have management priority in a given area. Differences in responses to different woodland growth types and woodland structural attributes therefore strongly suggest a need for spatial variation in management practices so that the different requirements of different species might be met in different parts of a given landscape. This is a common response in landscape approaches to biodiversity conservation: that is, “not to do the same thing everywhere” [88].

As outlined above, we have completed a major comparative study of bird responses to different revegetation growth types. Further key work that we have planned will include documenting longitudinal changes, such as those associated with woodland succession on bird assemblages. Presently the bird assemblages of plantings and resprout and seedling regrowth are markedly different (Figures 3 and 4) and therefore, a key question is whether the bird assemblages of these areas will eventually come to resemble one another or whether they will continue to be different and perhaps even diverge. This will be an important part of the continuation of sampling as highlighted by findings from other studies. For example, Wilkins et al. [89] evaluated the success of revegetation treatments on mined Australian coastal sand plains. They showed that revegetated areas were on a trajectory toward development of a new ecological community that differed significantly in species composition from pre-mining vegetation and adjacent un-mined vegetation. Thus, it will be important to maintain the work we have summarised in this paper as a true longitudinal study [90].

## Supporting Information

Appendix S1 **Vegetation attributes measured in different growth types (DOC).**
(DOC)

Appendix S2 **Percentage of sites with a particular growth type occupied by an individual species over the entire sampling period (DOC).**
(DOC)

Appendix S3 **Significant results from Steel-Dwass multiple comparisons (DOC).**
(DOC)
