# Supplementary material for: Not All Kinds of Revegetation Are Created Equal: Revegetation Type Influences Bird Assemblages in Threatened Australian Woodland Ecosystems
Source: PLoS One. 2012 Apr 6;7(4):e34527. doi: 10.1371/journal.pone.0034527 (PMC3320884; doi:10.1371/journal.pone.0034527)
Supplement: Appendix S1 — Vegetation attributes measured in different growth types (DOC). (DOC) [file pone.0034527.s001.doc]

**Not all kinds of revegetation are created equal. Revegetation type influences bird assemblages in threatened Australian woodland ecosystems**

D.B. Lindenmayer^1^, A.R. Northrop-Mackie^1^, R. Montague-Drake^1^, M. Crane^1^, D. Michael^1^, S. Okada^1^ and P. Gibbons^1^

^1^Fenner School of Environment and Society, The Australian National University, Canberra, Australian Capital Territory, Australia

Correspondence: [david.lindenmayer@anu.edu.au](mailto:david.lindenmayer@anu.edu.au)

**Appendix S**1. Vegetation attributes measured in different growth types

| **Measure** | **Description** | **Range of values** |
| --- | --- | --- |
| Plantation area in a 500m radius | Amount (ha) of planted vegetation (predominantly native) in a 500m radius of the site^A^ | 0-23.3 |
| Native veg area in a 500m radius | Amount (ha) of native woody vegetation in a 500m radius of the site^A^ | 0-62.9 |
| Cleared area a 500m radius | Amount (ha) of cleared land (i.e. now non-woody cover) in a 500m radius of the site^A^ | 15.61-78.12 |
| Paddock trees a 500m radius | Total number of paddock trees in a 500m radius of the site^A^ | 0-1.51 trees |
| Total stems | Number of stems across all vegetation > 1.5 m height (adjusted to ha)^B^ | 0-340 stems/ha |
| Overstorey % cover | % projected foliage cover of woody vegetation =>10 m in height^B^ | 0-76.67% |
| Midstorey % cover | % total cover of woody vegetation 2-10 m in height^B^ | 0-95% |
| Understorey % cover | % total cover of woody vegetation <2 m in height^B^ | 0-53.33% |
| Logs per ha | Number of logs > 10 cm diameter and 1 m in length (adjusted to ha)^B^ | 0-650 logs/ha |
| Trees greater than 50cm per ha | Number of trees with a diameter at breast height =>50 cm (adjusted to ha)^B^ | 0-100 |
| Hollow trees per ha | Number of trees with visible hollows in or overhanging the plot (adjusted to ha)^B^ | 0-83 |
| Mistletoe per ha | Number of mistletoe clumps (adjusted to ha)^B^ | 0-358 |
| Dieback score | Each plot scored according to amount of tree dieback across all trees (0=no dieback, 1=branch tips dead, 2=extensive defoliation, 3=epicormic growth, 4=tree death) | 0-3.6 |
| Percentage native tussock | % of native tussock grass | 0-44.58% |
| Percentage annual grasses | % of annual grasses | 0-94.6% |

1. Estimated using satellite imagery.
2. Average of three 20 X 20 m plots established around the 0 m , 100 m and 200 m post at each site.
3. Average of twelve 1 X 1 m plots. Each plot established at the corner of the 20 X 20 m plots (see above).
