# Supplementary material for: Not All Kinds of Revegetation Are Created Equal: Revegetation Type Influences Bird Assemblages in Threatened Australian Woodland Ecosystems
Source: PLoS One. 2012 Apr 6;7(4):e34527. doi: 10.1371/journal.pone.0034527 (PMC3320884; doi:10.1371/journal.pone.0034527)
Supplement: Appendix S2 — Percentage of sites with a particular growth type occupied by an individual species over the entire sampling period (DOC). (DOC) [file pone.0034527.s002.doc]

**Not all kinds of revegetation are created equal. Revegetation type influences bird assemblages in threatened Australian woodland ecosystems**

D.B. Lindenmayer^1^, A.R. Northrop-Mackie^1^, R. Montague-Drake^1^, M. Crane^1^, D. Michael^1^, S. Okada^1^ and P. Gibbons^1^

^1^Fenner School of Environment and Society, The Australian National University, Canberra, Australian Capital Territory, Australia

Correspondence: [david.lindenmayer@anu.edu.au](mailto:david.lindenmayer@anu.edu.au)

***Appendix S2. Percentage of sites with a particular growth type occupied by an individual species over the entire sampling period***

| **Common and Scientific Names** | **Resprout regrowth** | **Seedling regrowth** | **Old growth** | **Planting** | **p-value** |
| --- | --- | --- | --- | --- | --- |
| Australasian Pipit *Anthus novaeseelandiae* | 5.88% | 5.32% | 9.11% | 18.64%^H^ | <.0001* |
| Australian Magpie *Gymnorhina tibicen* | 100.00% | 100.00% | 99.82% | 99.12% | 0.0532 |
| Australian Raven *Corvus coronoides* | 81.45% | 87.45% | 86.87% | 84.43% | 0.1394 |
| Black-chinned Honeyeater^C^ *Melithreptus gularis* | 12.22%^H^ | 13.31%^H^ | 5.60% | 3.29% | <.0001* |
| Black-faced Cuckoo-shrike *Coracina novaehollandiae* | 36.20% | 30.80% | 29.77% | 28.29% | 0.1156 |
| Blue-faced Honeyeater *Entomyzon cyanotis* | 4.07% | 6.46% | 6.48% | 6.14% | 0.6031 |
| Brown Falcon *Falco berigora* | 4.52% | 3.80% | 3.85% | 5.48% | 0.6118 |
| Brown-headed Honeyeater *Melithreptus brevirostris* | 2.71% | 5.70% | 2.28% | 3.73% | 0.0719 |
| Brown Songlark *Cincloramphus cruralis* | 12.22% | 13.69% | 14.71% | 24.78%^H^ | <.0001* |
| Brown Treecreeper^C^ *Climacteris picumnus* | 42.08%^H^ | 43.35%^H^ | 28.90% | 3.51%^L^ | <.0001* |
| Buff-rumped Thornbill *Acanthiza reguloides* | 4.07% | 9.51%^H^ | 2.45%^L^ | 5.48% | 0.0001* |
| Cockatiel *Nymphicus hollandicus* | 10.41% | 15.59% | 17.86%^H^ | 9.87%^L^ | <.0001* |
| Common Blackbird *Turdus merula* | 0.45%^L^ | 0.38%^L^ | 2.63% | 10.75%^H^ | <.0001* |
| Common Bronzewing *Phaps chalcoptera* | 14.93% | 21.67% | 15.76% | 15.79% | 0.1274 |
| Common Starling *Sturnus vulgaris* | 42.99%^L^ | 49.81%^L^ | 70.58%^H^ | 55.04% | <.0001* |
| Crested Pigeon *Ocyphaps lophotes* | 46.15%^L^ | 54.75% | 57.27% | 60.96% | 0.0069* |
| Crested Shrike-tit^C^ *Falcunculus frontatus* | 24.43% | 25.10%^H^ | 18.21% | 13.60%^L^ | 0.0002* |
| Crimson Rosella *Platycercus elegans* | 17.65% | 21.67%^H^ | 11.56%^L^ | 14.47% | 0.0013* |
| Diamond Firetail^C^ *Stagonopleura guttata* | 9.50% | 8.37% | 4.55%^L^ | 8.55% | 0.0272* |
| Dollarbird *Eurystomus orientalis* | 0.90% | 2.28% | 2.63% | 1.32% | 0.2834 |
| Dusky Woodswallow^C^ *Artamus cyanopterus* | 19.46%^H^ | 17.87%^H^ | 12.43% | 3.73%^L^ | <.0001* |
| Eastern Rosella *Platycercus eximius* | 85.52% | 81.37% | 88.09%^H^ | 78.73%^L^ | 0.0001* |
| Eastern Yellow Robin^C^ *Eopsaltria australis* | 5.43% | 6.46%^H^ | 2.10% | 1.97% | 0.0011* |
| European Goldfinch *Carduelis carduelis* | 0.00% | 0.76% | 0.70% | 3.73%^H^ | <.0001*+ |
| Fairy Martin *Hirundo ariel* | 1.36% | 0.76% | 2.80% | 1.75% | 0.205 |
| Flame Robin^C^ *Petroica phoenicea* | 5.88% | 6.46% | 5.25%^L^ | 14.69%^H^ | <.0001* |
| Fuscous Honeyeater *Lichenostomus fuscus* | 6.79% | 7.60%^H^ | 4.20% | 1.75%^L^ | 0.0009* |
| Galah *Cacatua roseicapilla* | 78.28%^L^ | 84.41% | 93.70%^H^ | 84.21% | <.0001* |
| Golden Whistler *Pachycephala pectoralis* | 5.43% | 3.42% | 2.45% | 3.29% | 0.1907 |
| Grey Butcherbird *Cracticus torquatus* | 14.03% | 19.39%^H^ | 15.41% | 9.87%^L^ | 0.0017* |
| Grey-crowned Babbler^C^ *Pomatostomus temporalis* | 1.81% | 9.13%^H^ | 6.30% | 3.07% | 0.0002* |
| Grey Fantail *Rhipidura fuliginosa* | 3.17% | 6.46% | 4.20%^L^ | 10.75%^H^ | <.0001* |
| Grey Shrike-thrush *Colluricincla harmonica* | 63.80% | 59.70% | 50.09%^L^ | 63.82%^H^ | <.0001* |
| Hooded Robin^C^ *Melanodryas cucullata* | 5.88%^H^ | 3.04% | 2.45% | 0.88%^L^ | 0.0015* |
| Horsfield’s Bronze Cuckoo *Chrysococcyx basilis* | 7.69% | 6.08% | 5.43% | 8.55% | 0.1503 |
| Horsfield’s Bushlark *Mirafra javanica* | 0.45% | 1.14% | 2.10% | 2.85% | 0.1476 |
| House Sparrow *Passer domesticus* | 3.17%^L^ | 4.56%^L^ | 10.33% | 13.38%^H^ | <.0001* |
| Jacky Winter^C^ *Microeca fascinans* | 27.60%^H^ | 24.71%^H^ | 12.26% | 4.61%^L^ | <.0001* |
| Laughing Kookaburra *Dacelo novaeguineae* | 45.25% | 48.29% | 52.19%^H^ | 30.26%^L^ | <.0001* |
| Leaden Flycatcher *Rhipidura fuliginosa* | 1.81% | 3.04% | 1.40% | 0.88% | 0.203+ |
| Little Corella *Cacatua sanguinea* | 3.17%^L^ | 3.42%^L^ | 10.33%^H^ | 7.89% | 0.0002* |
| Little Friarbird *Philemon citreogularis* | 21.27%^H^ | 19.77%^H^ | 14.19% | 9.65%^L^ | <.0001* |
| Little Lorikeet *Glossopsitta pusilla* | 6.33%^H^ | 3.04% | 2.45% | 0.88%^L^ | 0.0006* |
| Little Raven *Corvus mellori* | 9.95% | 4.94% | 7.71% | 11.84%^H^ | 0.0287* |
| Magpie Lark *Grallina cyanoleuca* | 76.02% | 78.71% | 81.26% | 74.34% | 0.0332* |
| Masked Woodswallow^C^ *Artamus personatus* | 6.33% | 2.66% | 3.50% | 4.17% | 0.1057 |
| Mistletoebird *Dicaeum hirundinaceum* | 8.60% | 11.79%^H^ | 7.71% | 1.97%^L^ | <.0001* |
| Nankeen Kestrel *Falco cenchroides* | 1.36% | 1.52% | 1.58% | 2.63% | 0.6709+ |
| Noisy Friarbird *Philemon corniculatus* | 19.91% | 19.77% | 15.41% | 12.94% | 0.0987 |
| Noisy Miner *Manorina melanocephala* | 72.40% | 63.50% | 78.81%^H^ | 51.97%^L^ | <.0001* |
| Olive-backed Oriole *Oriolus sagittatus* | 4.98%^H^ | 4.94%^H^ | 1.75% | 0.22%^L^ | <.0001* |
| Pallid Cuckoo *Cuculus pallidus* | 6.33% | 7.22% | 4.38% | 2.85% | 0.0275* |
| Peaceful Dove *Geopelia striata* | 30.77%^H^ | 27.76% | 22.07% | 17.32%^L^ | 0.0002* |
| Pied Butcherbird *Cracticus nigrogularis* | 20.81% | 24.71% ^H^ | 24.17% | 12.06%^L^ | <.0001* |
| Pied Currawong *Strepera graculina* | 15.84% | 13.31% | 11.56% | 9.43% | 0.0359* |
| Rainbow Bee-eater *Merops ornatus* | 15.38% | 12.93% | 10.86% | 8.33% | 0.0325* |
| Red-browed Finch *Neochmia temporalis* | 1.81% | 0.76% | 0.88% | 3.07%^H^ | 0.0273*+ |
| Red-capped Robin^C^ *Petroica goodenovii* | 2.71% | 3.42% | 1.93%^L^ | 10.53%^H^ | <.0001* |
| Red-rumped Parrot *Psephotus haematonotus* | 66.06%^L^ | 82.13% | 79.51% | 77.41% | <.0001* |
| Red Wattlebird *Anthochaera carunculata* | 40.27% | 40.68% | 28.37%^L^ | 50.88%^H^ | <.0001* |
| Restless Flycatcher^C^ *Myiagra inquieta* | 24.43%^H^ | 16.73% | 11.73% | 7.89%^L^ | <.0001* |
| Rufous Songlark *Cincloramphus mathewsi* | 36.20% | 36.88% | 33.45% | 37.28% | 0.1868 |
| Rufous Whistler^C^ *Pachycephala rufiventris* | 18.10% | 21.67% | 14.89%^L^ | 28.51%^H^ | <.0001* |
| Sacred Kingfisher *Todiramphus sanctus* | 11.31% | 14.83%^H^ | 10.68% | 4.61%^L^ | <.0001* |
| Silvereye *Zosterops lateralis* | 0.90% | 2.28% | 1.05% | 3.51%^H^ | 0.0198* |
| Southern Whiteface^C^ *Aphelocephala leucopsis* | 3.17% | 5.32% | 2.45%^L^ | 5.92% | 0.0372* |
| Speckled Warbler^C^ *Chthonicola sagittata* | 1.81% | 2.66% | 0.35%^L^ | 4.17%^H^ | 0.0004* |
| Spotted Pardalote *Pardalotus punctatus* | 3.17% | 3.42% | 1.75% | 2.19% | 0.4146 |
| Striated Pardalote *Pardalotus striatus* | 66.06% | 68.82% | 77.06%^H^ | 56.14%^L^ | <.0001* |
| Stubble Quail *Coturnix pectoralis* | 3.17% | 4.18% | 4.20% | 6.80% | 0.1667 |
| Sulphur-crested Cockatoo  *Cacatua galerita* | 34.84% | 24.33%^L^ | 42.03%^H^ | 36.18% | <.0001* |
| Superb Fairy-wren *Malurus cyaneus* | 19.46%^L^ | 17.11%^L^ | 13.66%^L^ | 61.62%^H^ | <.0001* |
| Superb Parrot^C^ *Polytelis swainsonii* | 13.12% | 11.03% | 11.21% | 9.21% | 0.3281 |
| Tree Martin *Hirundo nigricans* | 2.71% | 6.84% | 6.48% | 2.41%^L^ | 0.0017* |
| Wedge-tailed Eagle *Aquila audax* | 0.90% | 2.28% | 2.98% | 1.75% | 0.2537 |
| Weebill *Smicornis brevirostris* | 13.57% | 17.87% | 10.68%^L^ | 16.89% | 0.0169* |
| Welcome Swallow *Hirundo neoxena* | 13.12%^L^ | 19.77% | 23.12% | 21.27% | 0.0176* |
| Western Gerygone *Gerygone fusca* | 3.62% | 6.46% | 2.28%^L^ | 6.58%^H^ | 0.0021* |
| White-browed Babbler^C^ *Pomatostomus superciliosus* | 4.52% | 8.37%H | 3.50% | 4.17% | 0.0175* |
| White-browed Woodswallow^C^ *Artamus superciliosus* | 22.17% | 16.35% | 16.81% | 14.47% | 0.0490* |
| White-naped Honeyeater *Melithreptus lunatus* | 6.79%^H^ | 6.08%^H^ | 3.15% | 0.44%^L^ | <.0001* |
| White-plumed Honeyeater *Lichenostomus pencillatus* | 72.40% | 77.57% | 67.08%^L^ | 85.09%^H^ | <.0001* |
| White-throated Gerygone *Gerygone olivacea* | 1.81% | 3.04% | 0.70% | 1.75% | 0.0825+ |
| White-throated Treecreeper *Cormobates leucophaeus* | 14.48%^H^ | 15.59%^H^ | 4.20%^L^ | 0.44%^L^ | <.0001* |
| White-winged Chough *Corcorax melanorhamphos* | 45.25%^H^ | 40.30% | 38.00% | 31.14%^L^ | 0.0017* |
| White-winged Triller^C^  *Lalage tricolor* | 19.46% | 20.91%^H^ | 13.84% | 12.50% | 0.0002* |
| Willie Wagtail *Rhipidura leucophrys* | 69.23%^L^ | 77.19% | 72.33%^L^ | 87.50%^H^ | <.0001* |
| Yellow-faced Honeyeater *Lichenostomus chrysops* | 4.07% | 3.04% | 2.80% | 1.97% | 0.506 |
| Yellow-rumped Thornbill *Acanthiza chrysorrhoa* | 15.38%^L^ | 22.43% | 20.32%^L^ | 39.47%^H^ | <.0001* |
| Yellow Thornbill *Acanthiza nana* | 3.62% | 6.84% | 4.03%^L^ | 14.25%^H^ | <.0001* |

A “C” denotes a bird that is of conservation concern. The Cochran-Mantel-Haenszel Chi-squared test was run for each species, stratified for year and season and we have presented the p-values from these tests. We deemed that P-values less than 0.05 corresponded to significant differences in species occurrence between growth types as denoted with a star (*). A cross (+) corresponds to species for which there was < 5 counts in a particular growth type and for which the chi-squared test and corresponding p-value may be unreliable. For the ANOM results, an “H” depicts the growth type which the species was found significantly (P<0.05) more on average in. Equivalently, an “L” represents a species that is found significantly less on average in that particular growth type.
