# Supplementary material for: Not All Kinds of Revegetation Are Created Equal: Revegetation Type Influences Bird Assemblages in Threatened Australian Woodland Ecosystems
Source: PLoS One. 2012 Apr 6;7(4):e34527. doi: 10.1371/journal.pone.0034527 (PMC3320884; doi:10.1371/journal.pone.0034527)
Supplement: Appendix S3 — Significant results from Steel-Dwass multiple comparisons (DOC). (DOC) [file pone.0034527.s003.doc]

**Not all kinds of revegetation are created equal. Revegetation type influences bird assemblages in threatened Australian woodland ecosystems**

D.B. Lindenmayer^1^, A.R. Northrop-Mackie^1^, R. Montague-Drake^1^, M. Crane^1^, D. Michael^1^, S. Okada^1^ and P. Gibbons^1^

^1^Fenner School of Environment and Society, The Australian National University, Canberra, Australian Capital Territory, Australia

Correspondence: [david.lindenmayer@anu.edu.au](mailto:david.lindenmayer@anu.edu.au)

**Appendix S3. Significant results from Steel-Dwass multiple comparisons**

|  | **Level** | **- Level** | **Mean Difference** | **Std Err Dif** | **Z** | **p-Value** |
| --- | --- | --- | --- | --- | --- | --- |
| Cleared area in a 500m radius | planting | resprout regrowth | 25.45 | 6.10 | 4.17 | 0.00 |
|  | planting | old growth | 17.86 | 6.79 | 2.63 | 0.04 |
|  | planting | seedling regrowth | 15.56 | 6.08 | 2.56 | 0.05 |
| Paddock trees in a 500m radius | planting | resprout regrowth | -17.55 | 6.10 | -2.88 | 0.02 |
|  | planting | seedling regrowth | -19.23 | 6.08 | -3.17 | 0.01 |
|  | planting | old growth | -27.09 | 6.79 | -3.99 | 0.00 |
| Total stems | planting | old growth | 54.19 | 6.79 | 7.98 | <.0001 |
|  | planting | resprout regrowth | 32.27 | 6.10 | 5.29 | <.0001 |
|  | natural regrowth | resprout regrowth | 16.77 | 4.56 | 3.68 | 0.00 |
|  | old growth | seedling regrowth | -38.23 | 6.40 | -5.97 | <.0001 |
| Overstorey % cover | planting | seedling regrowth | -39.03 | 5.86 | -6.66 | <.0001 |
|  | planting | resprout regrowth | -41.70 | 5.87 | -7.10 | <.0001 |
|  | planting | old growth | -60.36 | 6.71 | -8.99 | <.0001 |
| Midstorey % cover | planting | old growth | 62.57 | 6.69 | 9.35 | <.0001 |
|  | planting | resprout regrowth | 42.03 | 6.08 | 6.91 | <.0001 |
|  | planting | seedling regrowth | 27.75 | 6.07 | 4.57 | <.0001 |
|  | seedling regrowth | resprout regrowth | 19.22 | 4.44 | 4.33 | <.0001 |
|  | old growth | seedling regrowth | -33.38 | 6.16 | -5.42 | <.0001 |
| Understorey % cover | planting | old growth | 33.40 | 6.23 | 5.36 | <.0001 |
|  | planting | resprout regrowth | 15.76 | 5.92 | 2.66 | 0.04 |
| Logs per ha | old growth | resprout regrowth | 16.82 | 6.46 | 2.60 | 0.05 |
|  | planting | seedling regrowth | -39.69 | 5.86 | -6.78 | <.0001 |
|  | planting | resprout regrowth | -43.56 | 5.89 | -7.40 | <.0001 |
|  | planting | old growth | -64.23 | 6.72 | -9.56 | <.0001 |
| Trees greater than 50cm per ha | old growth | seedling regrowth | 33.92 | 6.40 | 5.30 | <.0001 |
|  | old growth | resprout regrowth | 22.40 | 6.46 | 3.47 | 0.00 |
|  | planting | resprout regrowth | -36.02 | 5.66 | -6.36 | <.0001 |
|  | planting | seedling regrowth | -43.27 | 5.77 | -7.49 | <.0001 |
|  | planting | old growth | -66.39 | 6.67 | -9.96 | <.0001 |
| Hollow trees per ha | old growth | seedling regrowth | 20.99 | 6.39 | 3.29 | 0.01 |
|  | old growth | resprout regrowth | 20.59 | 6.45 | 3.19 | 0.01 |
|  | planting | seedling regrowth | -31.02 | 4.96 | -6.26 | <.0001 |
|  | planting | resprout regrowth | -32.01 | 4.95 | -6.47 | <.0001 |
|  | planting | old growth | -59.64 | 6.42 | -9.29 | <.0001 |
| Mistletoe per ha | seedling regrowth | resprout regrowth | -11.92 | 4.52 | -2.63 | 0.04 |
|  | planting | seedling regrowth | -31.82 | 5.53 | -5.76 | <.0001 |
|  | planting | resprout regrowth | -42.72 | 5.65 | -7.55 | <.0001 |
|  | planting | old growth | -54.02 | 6.53 | -8.27 | <.0001 |
| % Annual grasses | old growth | seedling regrowth | 17.19 | 6.40 | 2.69 | 0.04 |
|  | planting | seedling regrowth | -16.34 | 5.15 | -3.17 | 0.01 |
| % Native tussock | planting | resprout regrowth | -15.31 | 5.09 | -3.01 | 0.01 |
|  | planting | seedling regrowth | -16.34 | 5.15 | -3.17 | 0.01 |

A positive mean difference shows that Level A has a significantly higher value than Level B. A negative mean difference shows that Level B is significantly higher than Level A.
